# Investigating parental care behaviour in same-sex pairing of zoo greater flamingo (*Phoenicopterus roseus*)

**DOI:** 10.7717/peerj.5227

**Published:** 2018-07-18

**Authors:** Barbara Regaiolli, Camillo Sandri, Paul E. Rose, Vittoria Vallarin, Caterina Spiezio

**Affiliations:** 1 Research & Conservation Department, Parco Natura Viva—Garda Zoological Park, Verona, Italy; 2 Department of Animal Health Care and Management, Parco Natura Viva—Garda Zoological Park, Verona, Italy; 3 College of Life & Environmental Sciences, University of Exeter, Exeter, United Kingdom; 4 Department of Neurosciences, University of Parma, Parma, Italy

**Keywords:** Female–female pairs, Zoo flamingo, Reproductive behaviour, Egg incubation

## Abstract

Same-sex pair bonds have been documented in several animal species and they are widespread in birds. However, little is known about the evolutionary origin and the adaptive value of such behaviour. The aim of this study was to investigate the parental behaviour of four zoo female greater flamingos involved in two breeding pairs, housed in a flock at Parco Natura Viva, Italy. Further, the behaviour of the study females was compared with that of male and female flamingos in heterosexual pairs described in a previous published work on this same flock. For each pair, the behaviour of both birds during the incubation period was recorded and twenty 10-minute sessions were run within the incubation period. A continuous focal animal sampling method was used to collect data on location (on the nest or not on the nest) and the parental care behaviour (e.g.: agonistic behaviours toward disturbing conspecifics, egg-care, nest-building, self-comfort behaviour, sleeping) of the two pairs. Data of the current study females were compared with those of females and males involved in heterosexual pairs of this same flock. Results showed that within each pair the egg-layer female stayed away from the nest more than the other female. In addition, the female that did not lay an egg was more involved in agonistic behaviour compared to other females, particularly when in specific locations. In heterosexual pairs, male flamingos were more involved in the incubation and in nest protection. Moreover, no significant differences in the time spent on the nest and away from the nest between the heterosexual male and the non-layer females of same-sex pairs were found. The same findings were reported when comparing heterosexual females and the egg-layer females of the same-sex pairs. Therefore, our findings suggest that in greater flamingos the behaviour of the female–female pairs seems to be equivalent to that of male-female bonds. Such research provides more insight into flamingo social behaviour, and their reproductive cycle, and provides information on why pair bonds may form and how these affect the wider breeding behaviour of the flock.

## Introduction

Despite the variety and widespread presence of same-sex sexual behaviour in animals, little is known about the evolutionary origin and, at least in some cases, the adaptive value of such behaviour (Bailey & Zuk, 2009). Explanations for same-sex sexual behaviour rely on both proximate mechanisms, such as genetic, neurological, hormonal and social foundations of same-sex interactions and the adaptive significance of this phenomenon (Bailey & Zuk, 2009). Different adaptive explanations have been proposed, varying according to factors such as species, individual age or kinship. Same-sex behaviour has been hypothesised to have a social function, such as strengthening social bonds and alliances between individuals as well as favouring social cohesion and reducing tension (Vasey, Bernard & Gauthier, 1998; Mann, 2006), increasing or diminishing intra-sexual conflict (Preston-Mafham, 2006; Vervaecke & Roden, 2006), practicing courtship, territory acquisition or mounting in juveniles (McRobert & Tompkins, 1988; King, 2006), indirectly inseminate females by depositing sperm on or in other males (Levan, Fedina & Lewis, 2009) and favouring alloparenting (Kuhle & Radtke, 2013; Kuhle & Brezinski, 2016). Moreover, based on the heterosexual deprivation hypothesis, as a result of the “prison effect”, a skewed sex ratio might lead to a lack of available opposite-sex partners and therefore favour same-sex pairings (Bonnet et al., 2016; Jankowiak et al., 2018). In socially monogamous species, such as the Laysan albatross (*Phoebastria immutabilis*), female–female pairings share parenting responsibilities, incubating the eggs and rearing the chicks, faring better than unpaired females (Bailey & Zuk, 2009). Thus, it is likely that same-sex pairings in this social context may increase fitness benefits, improving the importance of extra-pair copulations, especially when the sex-ratio is biased toward females (Nisbet & Hatch, 1999; Conover & Hunt Jr, 1984). Similarly, in feral pigeons (*Columbia livia f. urbana*), experimentally evoked female–female pairs have been found to raise offspring in the same way of heterosexual pairs. In particular, no differences in the fledgling mass growth between different pairs were found, suggesting that same-sex behaviour could be a better alternative to postponed breeding or raising chicks alone (Jankowiak et al., 2018).

As documented in several species, same-sex pairings are widespread in birds (Bagemihl, 1999; Bailey & Zuk, 2009; Sommer & Vasey, 2006). Specifically, female–female pair bonds are noted in a range of bird species including gulls, *Laridae* (Conover & Hunt Jr, 1984; Hunt Jr, & Hunt, 1977; Mills, 1991), Greylag Gander, *Anser anser anser* (Kotrschal, Hemetsberger & Weiss, 2006), Zebra Finches, *Taeniopygia guttata* (Tomaszycki & Zatirka, 2014), and Laysan Albatross,—in which up to 30% of the pairs were female only (Lindsay et al., 2008; for review see MacFarlane, Blomberg & Vasey, 2010). All the above-mentioned studies on same-sex pairs in monogamous birds focused mainly on reproductive behaviour, but few studies investigated differences and similarities between male–female and same-sex parental care behaviour (Elie, Mathevon & Vignal, 2011).

Flamingos (*Phoenicopteridae*) are highly gregarious birds that live and breed in large dense flocks (Pickering, Creighton & Stevens-Wood, 1992; Rose, 2017), often including thousands of pairs. As individual identification and close approach to birds might constrain systematic observation of flamingos, studying these species and collecting reliable scientific data on their behaviour in the wild can be difficult (Studer-Tiersch, 1975; Studer-Thiersch, 2000; King, 2000). Therefore, research on zoo colonies is both relevant and complementary to wild studies to improve our knowledge of flamingo ecology and behaviour (King, 2000; Hinton et al., 2013). Studying the behaviour of flamingos in the wild and in controlled environments is important to improve husbandry and breeding of these species (Melfi, 2009; Rose, Croft & Lee, 2014; Rose & Croft, 2015; Frumkin et al., 2016; Rose, 2017). More scientific data are needed to extend our knowledge on a flamingo’s requirements in captivity (Rose, Croft & Lee, 2014) as they, like other birds, are rather underrepresented in zoo science research.

Greater flamingos (*Phoenicopterus roseus*) are serially monogamous birds (Johnson & Cézilly, 2009) and both partners take part in nest building and share incubation duties, care and protection of the egg (Studer-Tiersch, 1975; Brown & King, 2005). Both parents care for the egg by moving and rotating it with their beaks (Studer-Tiersch, 1975). When one flamingo leaves the nest, the other one simultaneously climbs on the nest to incubate the egg (Studer-Tiersch, 1975). Even though both sexes incubate the egg, research suggests that male flamingos incubate more than females (King, 1994; Shannon, 2000; Brown & King, 2005; Sandri et al., 2017b). Moreover, both partners perform aggressive behaviour toward other flamingos that may be disturbing incubation (Studer-Tiersch, 1975; Hughes, 2015) but male birds have been found to be more involved in nest protection (Wood et al., 2017; Sandri et al., 2017b), suggesting that a female with a strong and fit male might be more successful in reproduction.

In zoos, same-sex pairs of flamingos might be five to six percent of the breeding pairs of the flock (Bagemihl, 1999). They are common among juvenile birds and among females, which are more prone to homosexual pairs than males (Brown & King, 2005). However, female–female pairs generally last no more than one season, whereas male-male pairs could be long-lasting (Brown & King, 2005). Same-sex pairs of flamingos have been found to perform nesting and parental behavioural patterns similar to male–female pairs and their reproductive success in terms of hatching and raising foster chick is the same as heterosexual breeding pairs (King, 1994; Shannon, 2000; Brown & King, 2005; King, 2006). As such, the common occurrence of intra-sexual pairings in flamingos is worthy of further assessment and investigation.

**Figure 1:**
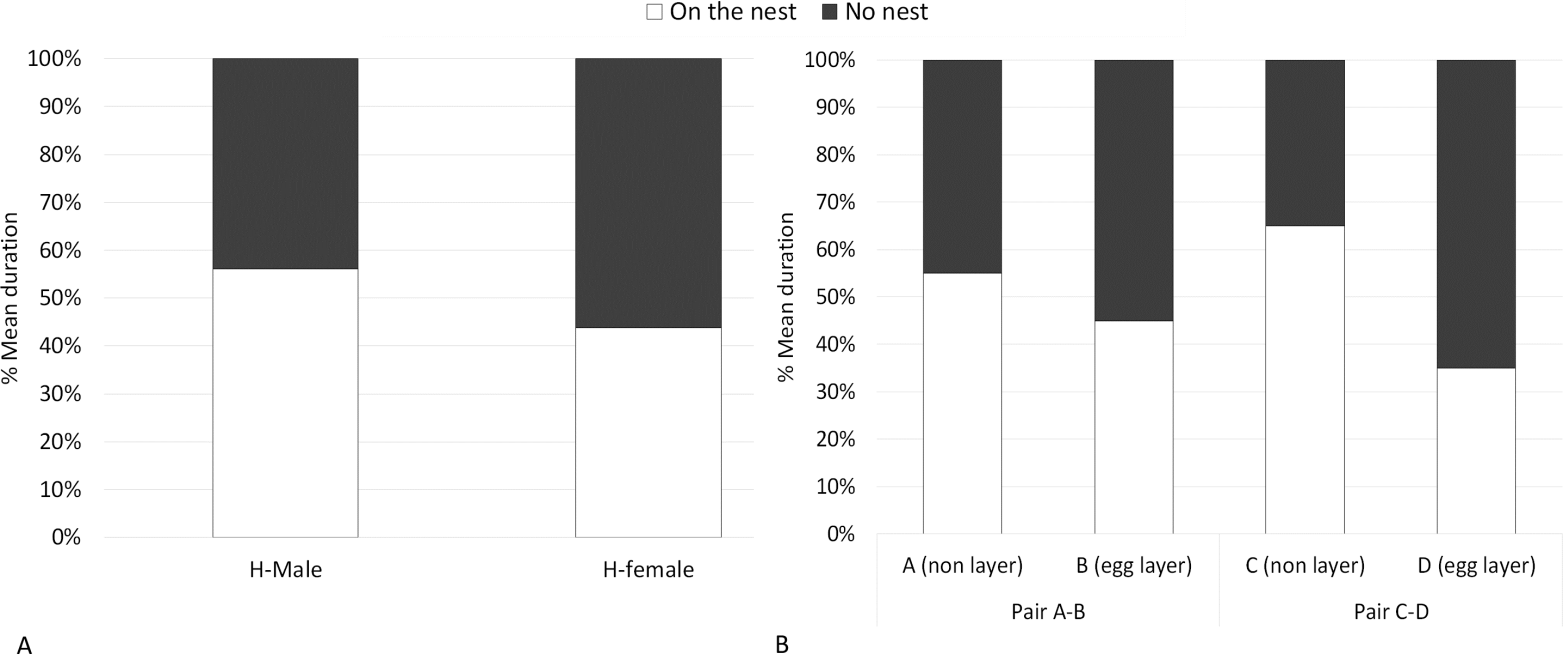
Locations of flamingos in heterosexual and in same-sex pairs. Percentage mean duration of time spent by flamingos on the nest and “no nest”. (A) Data from a previous study on heterosexual pairs in this same flock. (B) Data from the current study on the two same-sex pairs, A&B and C&D.

The current study focused on the parental behaviour of two female–female pairs of zoo greater flamingos (*Phoenicopterus roseus*). The aim of this study was to investigate the parental behaviour of these four female greater flamingos involved in two same-sex pairs, hosted in a flock at Parco Natura Viva, Italy. Behaviour patterns of these female–female pairs will be discussed around previous findings on parental care behaviours of female-male pairs of greater flamingos (Sandri et al., 2017b, see Fig. 1). According to previous literature on flamingo breeding reporting no differences between heterosexual and same-sex pairs’ parental care, we expected that behaviour patterns performed by the study flamingos during the incubation would be similar to those of males and females involved in female-male pairs (King, 1994; Shannon, 2000; Brown & King, 2005). Based on previous research on heterosexual pairs in this same study flock of greater flamingos in which male partners were more involved in incubation and nest protection, we expected differences between partners within the pair and similarities between partners of heterosexual and same-sex pairs (Sandri et al., 2017b, see Fig. 1). Furthermore, if parental care of same-sex pairs of greater flamingos would be equivalent to that of heterosexual pairs, we expect no significant difference between the non-layer females of same-sex pairs and males involved in heterosexual pairs. Similarly, no significant differences would be expected between the egg-layer females of same-sex pairs and females involved in heterosexual pairs.

## Materials & Methods

### Study subjects & area

Subjects of this study were two female–female pairs of greater flamingos, living in a flock composed of 147 individuals, 77 males and 70 females, housed in Parco Natura Viva—Garda Zoological Park, Italy. The age of the flamingos in the flock ranged from one to more than 20 years. The study was carried out between March and April 2016, during the peak of the flamingo breeding activity. For each pair, the behaviour of both females during the incubation period was recorded. Flamingos were housed in a 1,100 m^2^ enclosure composed of a muddy area and a grassy area. In the middle of the enclosure, there was a water pool with two islands, used by flamingos to build their nest mounds (nest density: about 2.5 nests/m^2^) and rear the chicks. The basal structure of the nest mound was built by humans, whereas flamingo pairs completed the nest using mud, soil and sand present in their enclosure. Food was provided to the flamingos once a day in an elongated feeding station. All subjects were parent-reared.

Flamingo identification was possible thanks to leg rings of different colour and three-letter combinations. Subjects of the study were two female–female pairs that incubated an egg in the 2016 breeding season. Female flamingos involved in the study were KUG (blue)-SRS (green) (first pair, referred to as A&B throughout the manuscript) and RHH (green)-HRY (red) (second pair, referred to as C&D throughout the manuscript). A and D were over 20 years old, whereas B and C were younger- hatched in 2010 and 2011 respectively. B had the same male partner from 2013 to 2015 and raised a chick in 2013 and in 2015 but had also produced several unhatched eggs. The female D had been with the same male partner from 2012 to 2015. Her eggs hatched successfully from 2012 to 2015, although in 2015 only the second egg that was laid hatched. Indeed, the first egg laid had to be taken away after the incubation period expired, to induce the female to lay a second egg (Pickering, 1992). In 2016, B and D laid the egg, with the other two females taking part in incubation after egg laying. In particular, at the beginning of the breeding season, these two females paired with their male partners of previous breeding seasons. However, after the egg-laying, the male flamingos left, and the females A and C replaced them, helping the two females to care of their eggs. The egg of the C&D pair hatched successfully, whereas the egg of the A&B pair failed to hatch.

### Procedure and data collection

Data collection started after egg-laying, when the male abandoned the female partner in the presence of the egg in the nest and a second female took part in the incubation process, forming the same-sex pair. For each pair, twenty 10-minute sessions were run during the incubation period. Two sessions per day were carried out; one in the morning (between 9:00 and 12:00) and one in the afternoon (between 14:00 and 17:00). The 20 observation sessions were carried out on random days within the incubation period. A continuous focal animal sampling method (Altmann, 1974) was used to collect durations of parental care behaviour of each partner of the two same-sex pairs. Data were collected using the focal sub-group sampling method in the presence of an egg on the nest. The chosen sub-groups were the same-sex pairs and the locations and postures of each partner within each pair were collected during the session. For each pair, we collected durations of individual and social behaviours, particularly parental care, performed by each partner within the pair when on the nest and near the nest. In addition, to be accurate, when one partner was not on the nest and was out of sight, the duration of “away from the nest” was also recorded (Sandri et al., 2017b). In other words, we did not collect the behaviour of the subject away from the focal point as “under most circumstances, the only condition under which such a record can be obtained is that in which all the individuals in the sample group are continuously visible throughout the sample period” (Altmann, 1974).

Data were collected on the location and posture of the birds in relation to the nest and the behavioural category performed. Regarding the location, we recorded whether each flamingo female was on the nest or not on the nest (referred to as “no nest” throughout the manuscript). When the females were on the nest, they could either be sitting (incubating) or standing. When the females were “no nest”, they could either be near the nest (less than 150 cm, which is approximately the highest flamingo body length; Del Hoyo, Elliott & Sargatal, 1992) or away from the nest (>150 cm), as previously mentioned. When flamingos were on the nest (both sitting and standing), we collected data on agonistic behaviour, such as extending the neck and beak at another bird (Stevens et al., 1992; Farrell, Barry & Marples, 2000), egg-care related behaviour (egg-rolling and moving), nest-building, self-directed comfort behaviour (preening, stretching and scratching) and sleeping (resting the head in the back). When flamingos were near the nest, all the other behaviours not directly associated with parental care were grouped in the behavioural category “Other” (for complete ethogram see Sandri et al., 2017b). As the observed behavioural categories were mutually exclusive, during focal-pair sampling we recorded the transition times, meaning that a behaviour ended when a new behaviour started.

### Comparison with heterosexual pairs

To test whether partners of same-sex pairs behaved similarly to males and females in heterosexual pairs, we compared data of the current study with those collected for heterosexual pairs of the same flock. We used the data on locations and postures in relation to the nest, in the presence of an egg, of 35 male–female pairs of flamingos. The data were collected by the same observer of the current study, using the same procedure and during the same time frame. Data collected for the 35 pairs were grouped and the percentage duration per session (ten sessions in total) of different locations and postures were calculated on the total observation time of the 35 pairs. In particular, the time spent in different locations and postures of each female of the same-sex pairs that laid the egg was compared with that of the group of heterosexual females (H-female). Similarly, the time spent in different locations and postures of each female that did not lay the egg was compared with that of the group of heterosexual males (H-male). We followed the single-case research design in which the behavior of a single subject is compared with a control group in order to assess behavioural similarities. Single-case research design has a high internal validity (Kratochwill et al., 2013; Scarpazza et al., 2016). In the current study, each female of the same-sex pairs was compared with a “control group” made of 35 male or female partners of the heterosexual pairs. In this study, a case-control design used in neuropsychology has been adapted and used to analyze the data. Case-control designs are studies “in which inferences concerning the cognitive performance of a single-case are made by comparing the case to a sample of healthy controls” (Crawford, Garthwaite & Ryan, 2011). The controls should be matched as much as possible (Crawford, Garthwaite & Ryan, 2011).

In the current study, for each session (ten sessions in total) we calculated the total duration of time spent in different locations and postures (on the nest standing and sitting, near the nest, away from the nest) by all females (*N* = 35) and all males (*N* = 35) of heterosexual pairs. Secondarily, for each session and for each sex, the percentages of time spent in each location and posture were calculated by dividing durations by the total observation time of all females and all males over all sessions (420,000 s). Following the same methodology, the percentages were calculated for the females involved in the same-sex pairs, A&B and C&D.

The study was carried out through the live observation of the birds, using non-invasive or stressful techniques. The study procedure was in accordance with the EU Directive 2010/63/EU and the Italian legislative decree 26/2014 for Animal Research. All procedures performed in the study were in accordance with the ethical standards of Parco Natura Viva as the research was approved by Parco Natura Viva ethical committee and by the local veterinary authority.

### Statistical analysis

A single-case analysis for single-case research design studies was performed to compare data between the two flamingo partners within each pair and the locations and postures between females of the same-sex pairs and males and females involved in heterosexual pairs (Barlow & Hersen, 1984; Jones, 2003; Scarpazza et al., 2016). Non-parametric statistic tests were used to analyse data (Box, Jenkins & Reinsel, 1994). Statistical significance was set at alpha = 0.05. Mann–Whitney tests were run to compare the duration of each location (near the nest, no nest), posture and behaviour between the females of each pair (Scarpazza et al., 2016; Sandri et al., 2017a) and to compare the time (%) spent in different locations and postures of females in same-sex pairs with those of greater flamingos involved in heterosexual pairs described in previous research on this flock (Fig. 1) (Sandri et al., 2017b). Bonferroni correction was used to adjust the *p*-value in the presence of multiple comparisons: male and female flamingos involved in heterosexual pairs were compared with the two non-layer and the two egg-layer females of the same-sex pairs respectively (two comparisons).

## Results

### Time spent in different locations

**Figure 2:**
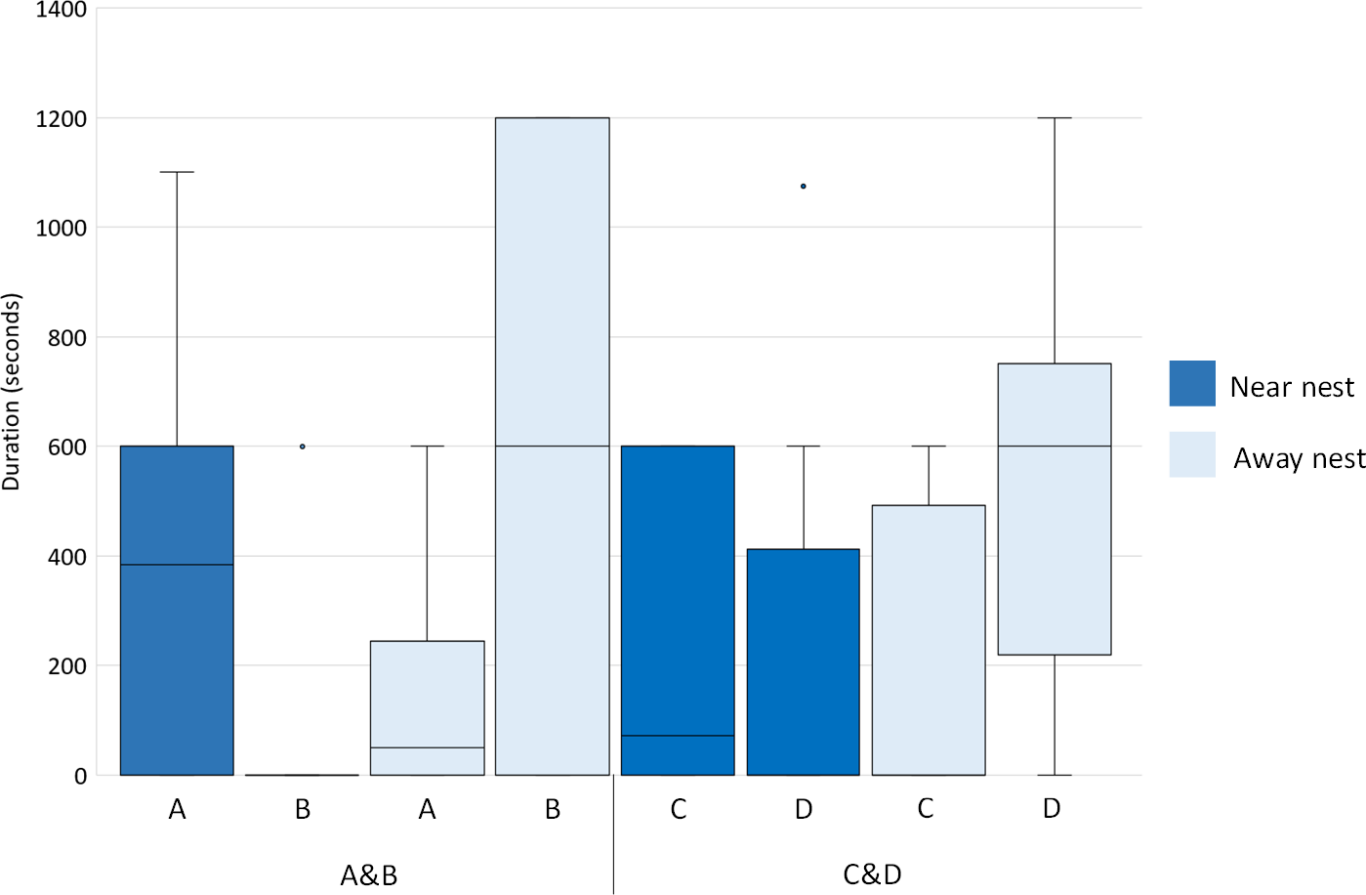
Locations of flamingos of two same-sex pairs. Time spent by female flamingos near the nest and away from the nest. Box and whisker plot of the time spent by flamingo females of the two pairs (A&B and C&D: B and D are the egg-layer females) near the nest (blue boxes) and away from the nest (pale blue boxes). The horizontal lines within the box indicate the medians, boundaries of the box indicate the first and third quartile. The whiskers extend up from the top of the box to the largest data element that is less than or equal to 1.5 times the interquartile range (IQR) and down from the bottom of the box to the smallest data element that is larger than 1.5 times the IQR. Values outside this range are considered outliers and are drawn as points.

Regarding the first pair, A&B, the female A (non-layer) spent 55% of the time on the nest (Fig. 1). The remaining time was spent “no nest” and included the locations near the nest and away from the nest. When “no nest”, A spent 33% of the time near the nest and 12% of the time away from the nest (Fig. 2). The female B (egg-layer) spent 45% of the time on the nest (Fig. 1); when “no nest”, B spent 5% of the time near the nest and 50% of the time away from the nest (Fig. 2). Regarding the second pair, C&D, the female C (non-layer) spent 65% of the time on the nest (Fig. 1); when “no nest”, C spent 21% of the time near the nest and 14% of the time away from the nest (Fig. 2). The female D (egg-layer) spent 35% of the time on the nest (Fig. 1); when “no nest”, D spent 18% of the time near the nest and 47% of the time away from the nest (Fig. 2). Some differences in the time spent near the nest and away from the nest were found within each pair. In particular, A (non-layer) was near the nest significantly more than B (egg-layer) (*U* = 21, *P* = 0.032, N_1_ = N_2_ = 10), whereas no significant differences between females were found in the time spent away from the nest (*U* = 24.5, *P* = 0.059, N_1_ = N_2_ = 10) (Fig. 2).

Regarding C&D, D (egg-layer) was away from the nest significantly more than C (non-layer) (*U* = 18.5, *P* = 0.019, N_1_ = N_2_ = 10) (Fig. 2), whereas no significant differences between females were found in the time spent near the nest (*U* = 44, *P* = 0.674, N_1_ = N_2_ = 10) (Fig. 2).

**Figure 3:**
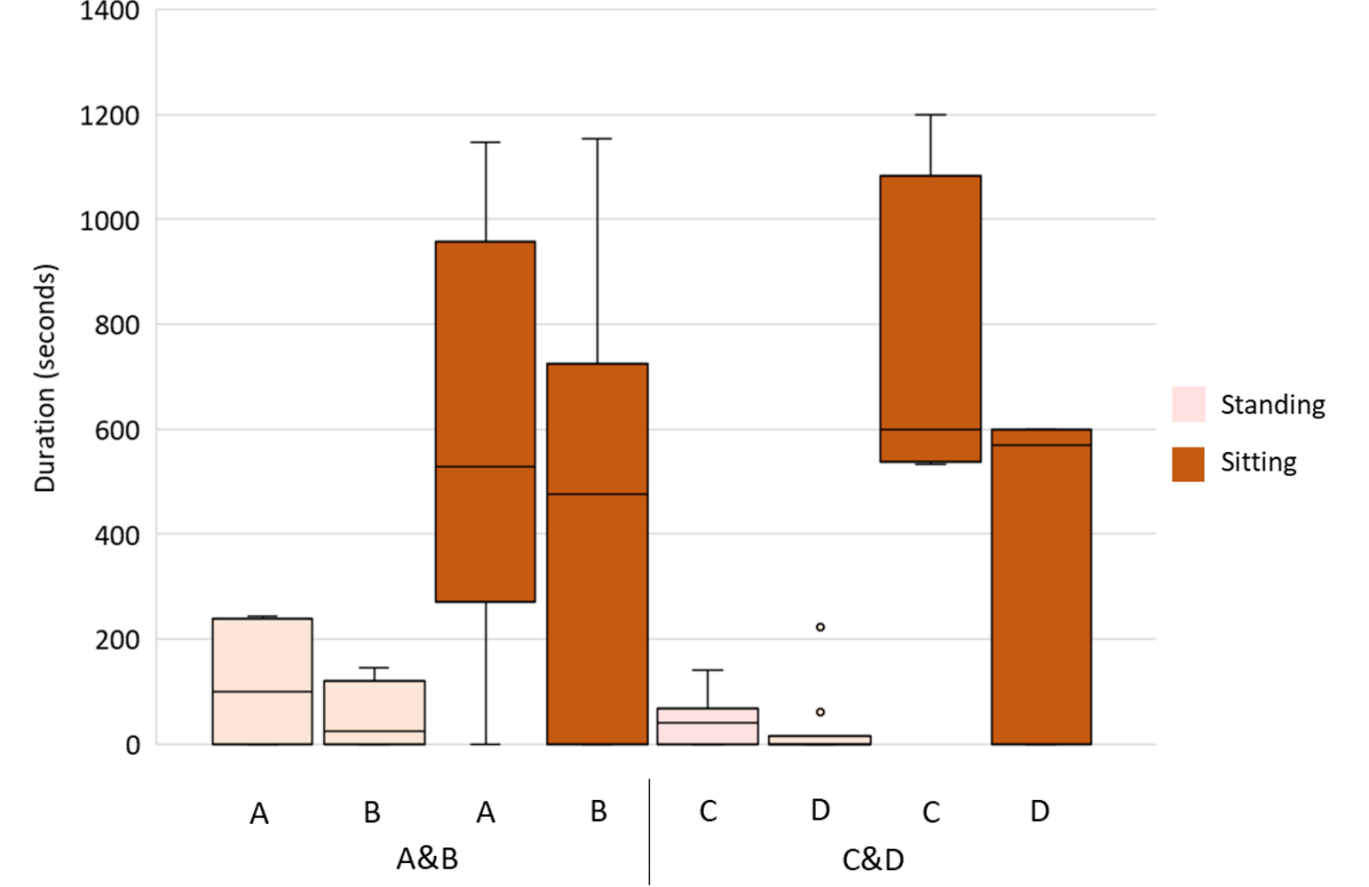
Postures on the nest of the partners of two same-sex pairs. Box and whisker plot of the time spent by flamingo females of the two pairs (A&B and C&D: B and D are the egg-layer females) on the nest standing (pale orange boxes) and sitting (orange boxes). The horizontal lines within the box indicate the medians, boundaries of the box indicate the first and third quartile. The whiskers extend up from the top of the box to the largest data element that is less than or equal to 1.5 times the interquartile range (IQR) and down from the bottom of the box to the smallest data element that is larger than 1.5 times the IQR. Values outside this range are considered outliers and are drawn as points.

### Time spent standing and sitting on the nest

Regarding flamingo postures on the nest, in pair A&B, the female A, the egg-layer, spent 9% of the observation time standing and 46% sitting, whereas B spent 4% of the time standing and 41% sitting (Fig. 3). In pair C&D, the female C, the egg-layer, spent 3% of the observation time standing and 62% sitting, whereas D spent 2% of the time standing and 33% sitting (Fig. 3). In both pairs, no significant differences between females were found in the time spent standing and sitting (A&B: standing: *U* = 35, *p* = 0.271; sitting: *U* = 48, *p* = 0.912. C&D: standing: *U* = 32, *p* = 0.187; sitting: *U* = 30.5, *p* = 0.150).

### Behaviours performed near the nest

When female flamingo partners were near the nest, they could perform agonistic behaviour, self-directed comfort behaviour, sleeping and other activities not directly associated with parental care (“other”). No significant differences were found within each pair in any behavioural category between the females A and B as well as between C and D (Table 1).

**Table 1:** Behavioural categories performed by female flamingos of the two pairs near the nest, on the nest (standing) and on the nest (sitting). For each female (A&B and C&D; B and D are the egg-layer females) the table reports the median (IQR) duration in seconds (s) of each behavioural category performed by the bird in different locations. Below each behavioural category are reported the Mann–Whitney *U* and *p* values from the comparison between the two females within each pair.

	Near the nest				On the nest (standing)			On the nest (sitting)				
	Agonistic	Comfort	Sleeping	Other	Agonistic	Comfort	Egg care	Agonistic	Comfort	Incubation	Nest building	Sleeping
A	4.00 (0–16) s	5.00 (0–258.25) s	0 (0–159.75) s	176.00 (0–335) s	0 (0–26) s	2.50 (0–17) s	88.00 (0–180.25) s	45.50 (0–99.5) s	0 (0–0) s	26.00 (0–126.25) s	375.50 (55.5–640.5) s	0 (0–0) s
B	0 (0–0) s	0 (0–0) s	0 (0–0) s	0 (0–0) s	0 (0–3.25) s	0 (0–2.5) s	23.00 (0–89) s	0.50 (0–65) s	0 (0–0) s	104.00 (0–315.5) s	36.00 (0–324.75) s	0 (0–145.75) s
*U*	25	31.5	35	24	42	37	35	41	45	39	27	35
*p*	0.064	0.174	0.271	0.054	0.569	0.347	0.271	0.522	0.728	0.43	0.089	0.271
C	0 (0–47)	1.5 (0–187.25) s	0 (0–0) s	0 (0–119.5) s	0 (0–0) s	0 (0–0) s	39.5 (0–67) s	76.50 (38.25–137.25) s	0 (0–0) s	22.50 (0–415.5) s	498.00 (289.5–571.75) s	0 (0–0) s
D	0 (0–9.5)	0 (0–87.5) s	0 (9–24.5) s	0 (0–67.25) s	0 (0–0) s	0 (0–1.25) s	0 (0–12.25) s	30.50 (0–36.5) s	0 (0–10.25) s	0 (0–451) s	33.00 (0–562.50) s	0 (0–0) s
*U*	39	38	45	45	40	44	28	16	35	45	30	50
*p*	0.430	0.384	0.728	0.728	0.472	0.674	0.103	0.011	0.271	0.728	0.142	0.968

### Behaviours performed on the nest

When female flamingo partners were standing on the nest, they could perform agonistic behaviour, self-directed comfort behaviour and egg-care. No significant differences were found in any behavioural category considering both the study pairs (see Table 1).

When female flamingo partners were sitting on the nest, they could perform agonistic behaviour, self-directed comfort behaviour, incubation, nest-building and sleeping. No significant differences between A and B (Table 1) were found in any behavioural category. Regarding the second pair, C performed significantly more agonistic behaviour than D (Mann–Whitney: *U* = 16, *P* = 0.011), whereas no other differences were found (see Table 1).

### Heterosexual pairs vs. female–female pairs

To evaluate whether greater flamingos in same-sex pairs behaved similarly to males and females involved in heterosexual pairs, we compared the time spent in different locations and postures by the non-layer and the egg-layer females with that of heterosexual males and females respectively (Table 2). Regarding the non-layer females, no significant differences were found between the H-male and both A and C for any of the locations and postures considered in the study, both on the nest and no nest (see Table 2 for median, IQR and *p*-values). Regarding the egg-layer females, the H-female spent significantly more time near the nest than the egg-layer B (Mann–Whitney: *U* = 10, *p* = 0.003) (see Table 2 for median and IQR). No other significant differences were found in any location and posture (see Table 2 for median, IQR and *p*-values).

**Table 2:** Time spent in different locations and postures by flamingos in heterosexual pairs and in female–female pairs. Median (interquartile range) duration of time (in seconds) spent in different locations and postures by male and female flamingos in heterosexual pairs (H-male and H-female) and by females of the same-sex pairs of the current study, A&B and C&D. For each location and posture, the medians are calculated on the total duration (%) of time spent per session (10 sessions in total). The H-male data have been compared with those of the non-layer females, A and C, whereas the H-female data have been compared with those of the egg-layer females. For each comparison, the table reports the *p*-values from the Mann–Whitney test. To correct for multiple comparisons (two comparisons), Bonferroni correction was used and the alpha value was set at 0.05∕2 = 0.025.

	H-Male	Non-layers		*U* and *p*-value	H-Female	Egg-layers		*U* and *p*-value
		A	C			B	D	
On the nest	5.57 (5.21–6.09) s	5 (3.75–10) s		*U* = 35, *p* = 0.271	4.43 (3.92–4.79) s	5 (0–6.25) s		*U* = 35, *p* = 0.271
			5 (5–10) s	*U* = 37, *p* = 0.347			5 (0–5) s	*U* = 37, *p* = 0.347
On the nest standing	0.19 (0.15–0.3) s	0.82 (0–1.99) s		*U* = 40, *p* = 0.472	0.23 (0.15–0.33) s	0.19 (0–0.99) s		*U* = 48, *p* = 0.912
			0.33 (0–0.56) s	*U* = 43, *p* = 0.624			0 (0–0.125) s	*U* = 20, *p* = 0.026
On the nest sitting	5.37 (5.02–5.88) s	4.40 (2.25–7.98) s		*U* = 34, *p* = 0.242	4.21 (3.65–4.53) s	3.98 (0–6.05) s		*U* = 49, *p* = 0.968
			5 (4.48–9.03) s	*U* = 37, *p* = 0.347			4.75 (0–5) s	*U* = 43, *p* = 0.624
No nest	4.43 (3.92–4.80) s	0 (6.25) s		*U* = 35, *p* = 0.271	5.57 (5.22–6.09) s	5 (3.75–10) s		*U* = 35, *p* = 0.271
			5 (0–5) s	*U* = 37, *p* = 0.347			5 (5–10) s	*U* = 37, *p* = 0.347
Near the nest	2.47 (2.22–2.89) s	3.20 (0–5) s		*U* = 40, *p* = 0.472	0.77 (0.55–1.12) s	0 (0–0)^*^ s		*U* = 10, *p* = 0.003
			0.60 (0–5) s	*U* = 40, *p* = 0.472			0 (0–3.44) s	*U* = 34, *p* = 0.242
Away from the nest	1.66 (1.44–2.22) s	0.42 (0–2.03) s		*U* = 31, *p* = 0.162	4.91 (4.24–5.23) s	5 (0–10) s		*U* = 50, *p* = 0.970
			0 (0–4.11) s	*U* = 30, *p* = 0.142			5 (1.82–6.25) s	*U* = 42, *p* = 0.569

**Notes.**

* Statistical significance: Bonferroni-corrected alpha-value  = 0.025.

## Discussion

Results from the current study underlined differences in parental care behaviour between the two female partners of the same-sex pairs, suggesting similarities with previous studies on greater flamingo heterosexual pairs (King, 1994; Shannon, 2000; Brown & King, 2005; Sandri et al., 2017b). Firstly, for both pairs, the egg layer spent more time not on the nest than the other female. Based on previous studies, male flamingos are expected to be more involved in incubation than females (Rendón-Martos et al., 2000; Rendón et al., 2014; Sandri et al., 2017b) (Fig. 1), presumably to allow the female to recover from the egg-laying effort and restock her reserves in case a new egg laying would be necessary (Jenni, 1974; Lenington, 1984; Cézilly, 1993; Reynolds & Szèkely, 1997; Johnson & Cézilly, 2009). Results from our study suggest that in female–female pairs the partner that did not laid the egg spent less time away from the nest and was more involved in parental and nest care than the female that laid the egg. Thus, the behaviour of the non-laying bird seemed to be similar to that of a male flamingo in a different-sex pair (Rendón-Martos et al., 2000; Rendón et al., 2014; Sandri et al., 2017b). Therefore, same-sex pairs might be important to improve breeding success of a species in the case of a pair breaking up, as the support of an unpaired bird to an individual that lost the partner after egg laying might compensate for its loss, favouring the standard parental care behaviour and therefore hatching success.

Regarding agonistic behaviour, the non-laying females were found to be more aggressive toward disturbing conspecifics than the other females. In particular, we found that C, the non-laying female of the second pair, while sitting on the nest showed significantly more agonistic behaviour than the egg-laying female in that pair, D, pecking and threatening other approaching flamingos. Male flamingos have been previously found to invest more time than females in nest and egg protection (Wood et al., 2017; Sandri et al., 2017b). Based on the study of Sandri et al. (2017b) on this same flamingo flock, male flamingos were significantly more aggressive than females when they were either near the nest and incubating the egg. Thus, the non-laying partner of female–female pairs seemed to show agonistic behavioural patterns similar to those reported in male flamingos in different-sex pairs (Johnson & Cézilly, 2009; Sandri et al., 2017b).

When female flamingos were standing on the nest, they could take care of the egg, moving or rotating it to improve the incubation effort. Within each study pair, no significant difference between the amount of time each partner spent caring for the egg was noted. The same findings have been found for female-male pairs of greater flamingos (Studer-Tiersch, 1975; Brown, Shannon & Farnell, 1983; Elphick, 2014; Sandri et al., 2017b). Therefore, we cautiously speculate on the presence of similarities in parental care behaviour between same-sex and female–male pairs. Incubation and egg care are important in determining the hatching success and it is possible that in heterosexual as well as in same-sex pairs both partners have to invest the same effort to maximize their reproductive success.

Our findings suggest that during the incubation period female flamingos involved in same-sex pairs displayed behavioural patterns similar to those of males and females involved in different-sex pairs (King, 1994; Shannon, 2000; Brown & King, 2005; Sandri et al., 2017b). The non-laying female partner seemed to be more involved in parental care and nest protection than the female that laid the egg and behaved like a male flamingo (Sandri et al., 2017b). All these findings are supported by the results from the comparisons of time spent in different locations and postures between same-sex pairs and heterosexual pairs of the same flock. Indeed, no significant differences between heterosexual males and the non-layer females A and C were found. The same finding was reported comparing the heterosexual females and the egg-layer females. Moreover, the egg-layer female B spent less time near the nest even than the heterosexual female and both females were near the nest less than males. Thus, the non-layers and the egg layers in same-sex pairs seemed to behave similarly to male and female in heterosexual pairs. Previous research on monogamous birds such as Zebra Finch (*Taeniopygia guttata*) and feral pigeons underlined that same-sex pair bonds might be equivalent to male–female bonds in socially monogamous species (Elie, Mathevon & Vignal, 2011; Jankowiak et al., 2018). In socially monogamous birds, having a partner might be advantageous for survival and reproductive success and same-sex pairing could therefore be important due to the need of a partnership (Elie, Mathevon & Vignal, 2011). Thus, our study seems to add to the body of evidence that same-sex pairings may be adaptive as they could favour allo-parenting behaviour, representing a mechanism to obtain allomothering investment from an unrelated female (Kuhle & Radtke, 2013; Kuhle & Brezinski, 2016).

Based on the heterosexual deprivation hypothesis, the occurrence of same-sex pairings might result from a lack of available opposite-sex partners (Bonnet et al., 2016; Jankowiak et al., 2018). However, the composition of the sub-group of unpaired flamingos of the study flock (*N* = 77) seems not to support this hypothesis, as the sex-ratio of the potential breeders was balanced. Indeed, when considering the age of the flamingos involved in heterosexual breeding pairs, potential breeders that were not involved in pair formation have been identified as females born before or in 2014 and males born before or in 2013. Among these birds, including the four females of the study same-sex pairs, the number of females was 23 whereas the number of males was 24, underlining a highly balanced sex-ratio.

Finally, we reported an overall absence of significant differences between the parental behaviour of males and females of heterosexual pairs and that of the non-layer and the egg-layer females of the same-sex pairs respectively. This result seems to support the presence of similarities in parental care behaviour between heterosexual and same-sex pairings of greater flamingos.

Findings of the current study seem to add to previous research on social monogamous birds, highlighting that same-sex pairing might be relevant for greater flamingo reproduction, as in this species both partners seem to play an important role in parental care behaviour and egg incubation.

## Conclusion

Results of this study on parental behaviour in female–female pairs of greater flamingos add to previous literature on same-sex pairings in birds and are useful to advance our knowledge of the behaviour of this species. In particular, this research suggests that same-sex pairing might be a compensation mechanism to improve the breeding success of greater flamingos, for example in the case of a pair breaking up. Thus, our study adds consistency to the alloparenting hypothesis, suggesting that fluid sexuality of females may be a mechanism to possess allomothering investment from a female not related to the offspring (Kuhle & Radtke, 2013; Kuhle & Brezinski, 2016; Bailey & Zuk, 2009). Similarly, our findings add to the body of evidence that same-sex pairings may improve fitness benefits (e.g., number of chicks raised successfully), reducing the number of unpaired females and increasing that of reared chicks (Nisbet & Hatch, 1999; Conover & Hunt Jr, 1984; Bailey & Zuk, 2009; Jankowiak et al., 2018). Finally, results of this study could advance our understanding of same-sex pairings among social monogamous birds, helping informed management decisions in widespread zoo flocks of this species.

##  Supplemental Information

10.7717/peerj.5227/supp-1 Supplemental Information 1 Raw data

## Additional Information and Declarations

### Data Availability

The following information was supplied regarding data availability: The raw data are included in a Supplemental File.
